# Cia Zeaxanthin Biosynthesis, *OsZEP* and *OsVDE* Regulate Striped Leaves Occurring in Response to Deep Transplanting of Rice

**DOI:** 10.3390/ijms23158340

**Published:** 2022-07-28

**Authors:** Qianyi Hao, Guangwang Zhang, Xilong Zuo, Ying He, Hanlai Zeng

**Affiliations:** 1 MOA Key Laboratory of Crop Ecophysiology and Farming System in the Middle Reaches of the Yangtze River, College of Plant Science and Technology, Huazhong Agricultural University, Wuhan 430070, China; haoqy@webmail.hzau.edu.cn (Q.H.); zhanggw@webmail.hzau.edu.cn (G.Z.); zuoxl@webmail.hzau.edu.cn (X.Z.); 2 Center of Crop Nanobiotechnology, Huazhong Agricultural University, Wuhan 430070, China

**Keywords:** rice, striped leaf occurrence, UPLC–MS/MS, zeaxanthin, *OsZEP*

## Abstract

The rice leaf color mutant B03S was previously generated from the photoperiod- and thermo-sensitive genic male sterile (PTGMS) rice line Efeng 1S, of which male sterility manifests by photoperiod and temperature but exhibits mainly temperature-sensitive characteristics. After these plants were deeply transplanted, the new leaves manifested typical zebra stripe patterns. Here, B03S was subjected to deep and shallow transplanting, shading with soil and aluminum foil, and control conditions in situ to determine the cause of the striped-leaf trait. The direct cause of striped leaves is the base of the leaf sheath being under darkness during deep transplanting, of which the critical shading range reached or exceeds 4 cm above the base. Moreover, typical striped leaves were analyzed based on the targeted metabolome method by ultra-performance liquid chromatography/tandem mass spectrometry (UPLC–MS/MS) combined with transcriptome and real-time quantitative PCR (qPCR)-based verification to clarify the metabolic pathways and transcriptional regulation involved. Carotenoids enter the xanthophyll cycle, and the metabolites that differentially accumulate in the striped leaves include zeaxanthin and its derivatives for photooxidative stress protection, driven by the upregulated expression of *OsZEP*. These findings improve the understanding of the physiological and metabolic mechanisms underlying the leaf color mutation in rice plants, enrich the theoretical foundation of the nonuniform leaf color phenomenon widely found in nature and highlight key advancements concerning rice production involving the transplanting of seedlings or direct broadcasting of seeds.

## 1. Introduction

Photosynthesis is a key energy transformation and physiological and biochemical process driving plant growth and development [1]. Leaves are the main organs for plant photosynthesis. Leaf color affects photosynthetic efficiency, reflecting the photosynthate assimilation ability [2,3]. Uneven green-colored leaves or leaf color mutations are common phenomena in plants and are of great value for studying photosynthetic pigment function and chloroplast development [4]. Rice (*Oryza sativa* L.) is one of the most important food crop species in the world and contributes greatly to the worldwide staple food supply. For the key goal of increasing rice yields per unit area, increasing biomass accumulation by improving photosynthetic efficiency is an effective measure [5]. Studying the mechanism of leaf color mutations has theoretical and practical importance in plants, especially in rice [6]. Plant leaf color is mainly determined by the types, contents, and proportions of pigments, such as chlorophyll, carotenoids, and anthocyanins [7,8,9,10]. Carotenoids often significantly change in albino or yellow leaves. The previous research shows that the total carotenoid contents are reduced in the leaves of albino rice compared with rice mutants [11]. Carotenoids participate in photomorphogenesis and photoprotection and are also considered signaling molecules that sense environmental stimuli for the regulation of plant growth and development [12]. Downstream of the carotenoid metabolic pathway, abscisic acid (ABA) is synthesized from neoxanthin by 9-cis-epoxycarotenoid dioxygenase (NCED) and carotenoid-cleaving dioxygenase (CCD), which together are key enzymes for plant stress signals biosynthesis in the regulation of physiological processes under environmental stress conditions [13].

The carotenoid metabolic pathway is widely reported to be regulated by light [14]. Generally, the carotenoid metabolic pathway can be divided into four stages, β-carotene biosynthesis, ABA biosynthesis, xanthophyll cycle, and lutein biosynthesis. The flow direction of metabolites is determined as described below. In the β-carotene biosynthesis pathway, the initial metabolite geranylgeranyl diphosphate (GGPP) is converted to β-carotene; From the intermediate metabolite lycopene, metabolites flow to two metabolic branches of lutein and ABA synthesis. Before ABA synthesis, there were three main types of carotenoids: violaxanthin, antheraxanthin, and zeaxanthin in the xanthophyll cycle, and the xanthophyll cycle is considered to have an important function in protecting photosynthetic organs from excess photooxidation, mainly through two ways: an inhibitory mechanism for thermal dissipation and a removal mechanism for phototoxic products [15,16,17,18]. Between the two defense strategies, zeaxanthin plays a key role in resisting light stress and ensuring normal plant growth [19,20]; this process is regulated by two key enzymes, zeaxanthin epoxidase (ZEP) and violaxanthin de-epoxidase (VDE) [21]. Under excess light energy, VDE activity is improved by acidification of the thylakoid lumen, resulting in the de-epoxidation of violaxanthin to antheraxanthin and subsequent zeaxanthin generation and accumulation [22]. Zeaxanthin can remove excess reactive oxygen species (ROS) in leaves [23].

The photoperiod- and thermo-sensitive genic male sterile (PTGMS) rice lines are used to produce hybrid seeds in two-line hybrid rice systems. Male sterility of PTGMS rice is caused by environmental factors of photoperiod and temperature. A natural mutant with striped leaves, B03S, was found by our research team from among the offspring of the PTGMS rice line Efeng 1S. This leaf color phenomenon of B03S ensures the identification of parental purity as quickly, conveniently, and simply as possible during the rice breeding process. Moreover, B03S is sensitive to transplanting depth, and the impact of transplanting depth on seedlings can be obviously reflected in phenotypic changes. Therefore, an intensive study of this material can also provide theoretical guidance for technical parameters of rice production, for example, a suitable depth for rice growth and development in actual agricultural operations involving mechanical transplanting and seed broadcasting.

Nonuniform-leaf color phenomena of plants are common in nature. Studying the reason underlying leaf color mutations is important for exploratory purposes [24,25,26]. The leaf color mutant B03S is an ideal material for researching the relevant physiological and biochemical processes. Although the genetic physiological basis of B03S has been studied relatively in-depth, the specific leaf color regulatory mechanism and key regulatory metabolites are unknown. We have previously found that the zebra leaf chloroplast growth and development status were abnormal, with obvious structural defects and abnormal photosynthetic pigment metabolism, especially the carotenoids. Therefore, we speculated that the carotenoid metabolism pathway and its metabolites might play an important role in the formation of its zebra leaves and carried out to excavate and verify the metabolic pathway sites and functional metabolites acting during the formation through this study by liquid chromatography/tandem mass spectrometry (LC–MS/MS), transcriptome analysis, and real-time quantitative PCR (qPCR) validation. The results indicate specific metabolic roles of the carotenoid pathways in the striped leaves of B03S, with regulatory zeaxanthin accumulating as a result of *OsZEP* and *OsVDE* expression for striped-leaf photooxidative stress protection. When taken together, these findings provide new insight into resolving the metabolic mechanisms underlying uneven leaf color distribution.

## 2. Results

### 2.1. Striped Leaves Phenotype of B03S Occurs in Response to Deep Transplanting

**Figure 1:**
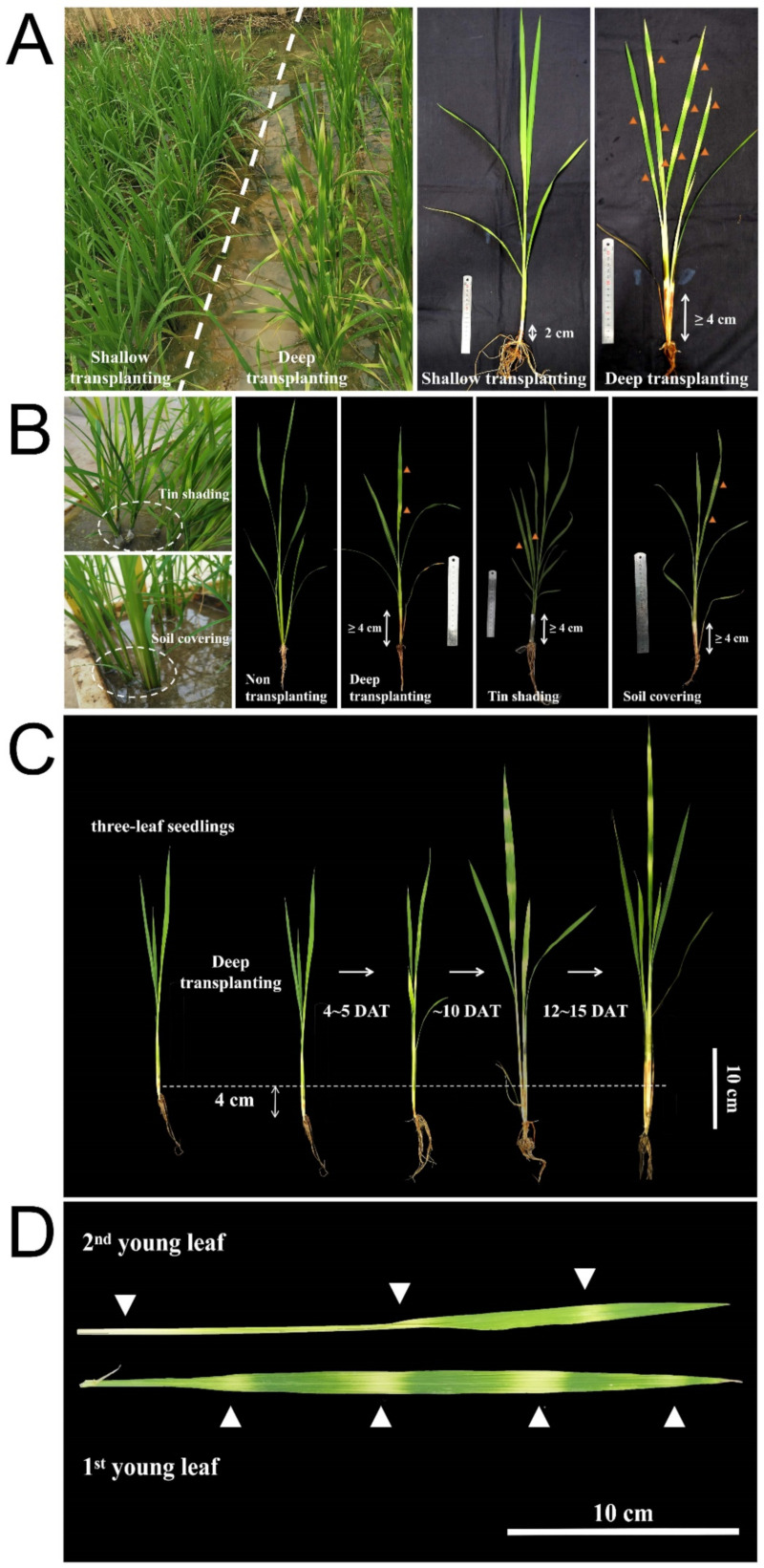
Phenotype of obvious striped leaves occurred in B03S under different treatments. (**A**) Deep/shallow transplanting plants in the field. (**B**) Soil covering and aluminum shading plants in pots. (**C**) The zebra stripes of leaves occurred under deep transplanting. (**D**) The obvious yellow-green stripes varied in different leaves of an individual plant at the same growth periods. Triangle symbols indicate significant the yellow stripes in leaves. DAT, day after transplanting.

In order to obtain plants with striped leaves, B03S seedlings at the 3-leaf stage were subjected to four different leaf sheath shading treatments, namely, shallow/deep transplanting and the soil/aluminum foil covering and nontransplanted CK (Figure 1A,B, Table 1, Supplementary Figure S1). The zebra stripes appeared on the new leaves of the plants in three groups, namely, the deep transplanting group and the soil/aluminum covering groups, after 4–5 days of treatment. The zebra stripe trait of B03S manifested as leaf sheath bleaching and a transverse and intermittent yellow-green stripe formation on the new leaves (Figure 1C,D). In the nontransplanting and shallow transplanting groups, there was no loss of green color in the leaf sheaths or leaves of B03S. When taken together, these results showed that the zebra-stripe leaf pattern that arises during rice seedling transplantation would be insufficient light for the leaf sheath at a certain range from the base of the plant to a height of 4 cm above the ground.

The extent of the yellow-green stripes of B03S induced under different shading treatments showed differences among the three groups (Supplementary Figure S1). The degree of stripes induced by deeply transplanting was significantly higher than that induced by in situ soil covered with soil and aluminum foil. In addition, the stripe characteristics appeared on both the leaf sheaths and the new leaves after deep transplanting, while those in response to shading with soil or aluminum foil appeared only on the leaf sheaths and the new leaves. The results of the above experiments showed that the main cause of the striped-leaf phenotype was not rooted damage caused by transplanting but, rather, insufficient light reaching the leaf sheath at a certain height. This mutant trait caused by environmental change is stably inherited in subsequent generations, indicating that this trait is a new mutant material that formed due to genetic change.

### 2.2. Metabolomics Analysis of the Leaf Colored Stripe Trait of B03S Targeted in the Carotenoid Pathway

**Figure 2:**
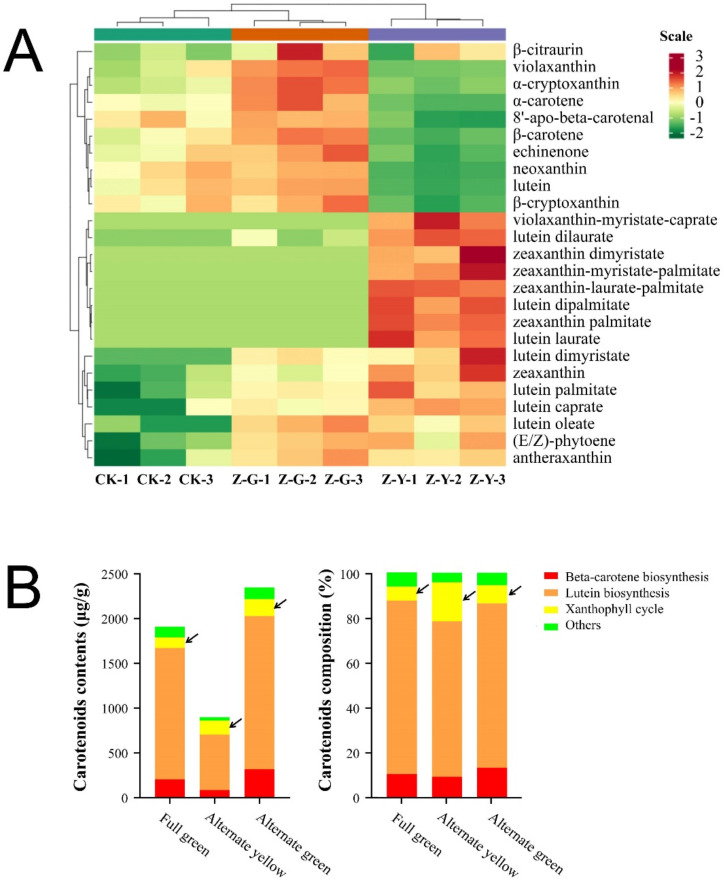
Differential analysis of carotenoids in the striped and green leaves of B03S. (**A**) Heatmap analysis of the main differentially accumulated metabolites of the carotenoid pathway. (**B**) The main differentially accumulated metabolites in different metabolic branching of the carotenoid pathway. CK, fully green leaves; Z-G, zebra-green striped in leaves/alternate green leaves; Z-Y, zebra-yellow striped in leaves/alternate yellow leaves. The arrow points to Related metabolites contents related to xanthophyll cycle.

Qualitatively and quantitatively targeted metabolomic analysis of carotenoids in B03S leaves was carried out through ultra-performance liquid chromatography/tandem mass spectrometry. In the zebra-yellow striped (Z-Y)/zebra-green striped (Z-G) parts and the fully green (CK) parts of the leaves of B03S, a total of 25 differentially accumulated metabolites were detected in the carotenoid metabolome (Figure 2A). The concentrations of carotenoids in the Z-Y stripes were significantly lower than those in the CK or Z-G stripes, with a proportion of only 38.2–47.10% in the CK and Z-G stripes (Figure 2B). Among the metabolites detected, the six predominant metabolites were lutein, β-carotene, antheraxanthin, zeaxanthin, violaxanthin, and neoxanthin, the contents of which were 617.88–1703.12, 78.81–310.94, 27.65–52.46, 9.43–43.44, 63.59–111.20, and 35.40–124.91 μg/g, respectively.

Of the twenty-five differentially accumulated metabolites, sixteen and nine were increased and decreased, respectively, in the Z-Y stripes (Figure 2A). Generally, these increased and decreased metabolites were the trace and predominant components, respectively. However, all eighteen differentially accumulated metabolites in the Z-G stripes were increased. Thus, the total contents of carotenoids in the Z-Y stripes were still decreased, and those in the Z-G stripes were substantially increased (Figure 2B).

To identify typical metabolites underlying the leaf color, nine differentially accumulated metabolites were chosen according to their content (≥1.00 μg/g) and the degree of accumulation (fold change ≥1.7 or ≤0.6). There were eight, three, and seven metabolites that significantly differed in Z-Y and CK, Z-G and CK, or Z-Y and Z-G pairwise comparison, respectively (Supplementary Figure S2). Notably, for the Z-Y stripes of B03S leaves, only one typical differentially accumulated metabolite was identified, zeaxanthin, with a content of 43.44 μg/g, which increased by 4.61-fold and 1.73-fold, respectively, compared with the contents of the samples from the fully-green CK leaves and Z-G stripes (Figure 2A). In addition, specific metabolites of xanthin ester derivatives were identified as being present in trace amounts only in the Z-Y leaves and not in the Z-G or CK leaves. Except for the typical and specific metabolites identified in the yellow stripes of the striped leaves, five specific metabolites were also identified: violaxanthin-myristate-caprate, lutein laurate, and lutein dipalmitate were specifically in the Z-Y laves, and lutein dilaurate and lutein dimyristate were in the Z-G and Z-Y leaves (Figure 2A).

In the green-colored stripes of striped leaves, most differentially accumulated metabolites were increased and mainly enriched in the lutein synthesis pathway (Figure 2B). In the yellow-colored ones, two metabolites, namely, antheraxanthin and zeaxanthin, were significantly increased, which was achieved via concentration of the metabolic flow, with the abundance of the metabolites increasing by 181.59% in the xanthophyll cycle, compared with 11.34%, 10.48%, and 30.57% of decreased metabolites in pathways β-carotene biosynthesis, lutein biosynthesis, and others, respectively (Figure 2B). The three major compounds in the xanthophyll cycle are violaxanthin (V), flaxanthin (A), and zeaxanthin (Z). The degree of lutein de-epoxidation indicated by the ratio of (Z + A) to (Z + A + V) in striped leaves was generally higher than that in the normal green leaves, which is also higher than that in Z-G leaves (Table 2).

### 2.3. Transcriptome Sequencing Analysis of the Leaf Color Stripe Trait of B03S

**Figure 3:**
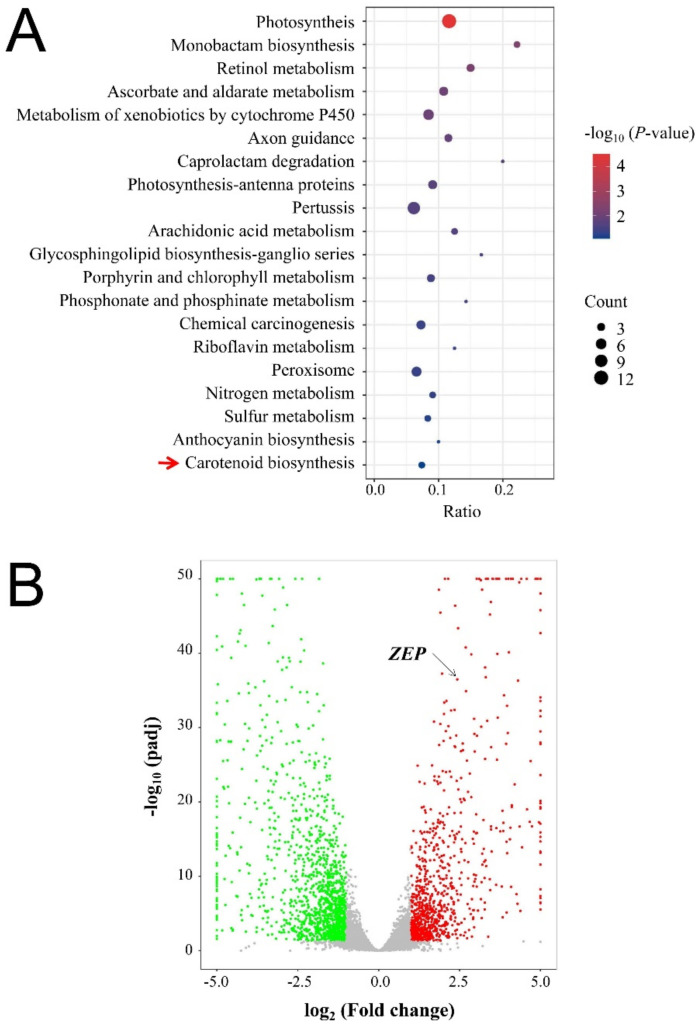
Metabolic pathway analysis of differentially expressed genes identified in the transcriptome. (**A**) Bubble diagram analysis of differentially gene expressions in metabolic pathway. (**B**) Volcano map of differentially expressed genes. Data are normalized and transformed of gene expression in zebra-green striped leaves and zebra-yellow striped leaves (*n* = 3). The red arrow points to the Carotenoid biosynthesis, and the black arrow points to the *ZEP*.

According to the regulation of the carotenoid pathway during the emergence of striped leaves, transcriptome analysis of the striped leaves of B03S was performed to further elucidate the molecular basis. The results showed that 21,570 genes, which overlapped with 89.1% of the rice genome, were co-expressed in the Z-G and Z-Y stripes of the B03S leaves, of which 1266 genes (accounting for 5.2%) and 1370 genes (accounting for 5.7%) were specifically expressed in those parts. Among them, the significantly differentially expressed genes (DEGs) between the Z-G and Z-Y leaves were selected according to the criteria of an FC ≥ 2/≤ 0.5 and a *p*-value ≤ 0.05. A total of 720 DEGs (303 upregulated and 417 downregulated) were identified in the Z-Y stripes of the B03S leaves compared with the Z-G leaves. According to the results of our Kyoto encyclopedia of genes and genomes (KEGG) analysis, these DEGs were significantly enriched in four common terms: photosynthesis, biosynthesis of secondary metabolites, ABA biosynthesis, and carotenoid biosynthesis (Figure 3A). Among them, 12 DEGs regulated the photosynthetic process; 43 DEGs were enriched in the secondary metabolic synthesis pathway, which involves sugar metabolism, phenylpropane metabolic processes, and chloroplast development (Supplementary Table S1). Among these DEGs, we found that chloroplast development genes (LOC_Os09g12660, LOC_Os03g20700, and LOC_Os03g52170), cytochrome P450 genes (LOC_Os11g41710, LOC_Os11g41680, and LOC_Os09g10340), phenylpropane metabolic pathway genes (LOC_Os09g25150, LOC_Os03g60509, and LOC_Os10g17260) and sugar metabolism genes (LOC_Os04g43390) were significantly upregulated in Z-G, compared with Z-Y (Supplementary Figure S3).

**Figure 4:**
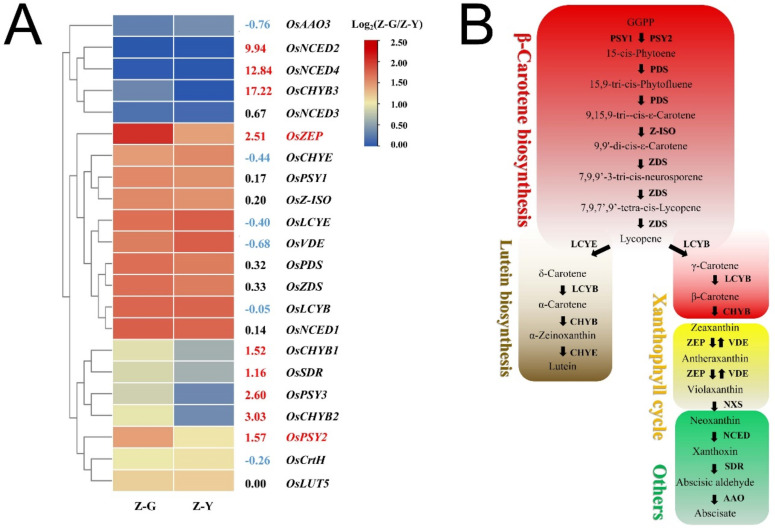
Transcriptome analysis of gene expressions involved in carotenoid biosynthesis-related pathways in the striped leaves of B03S. (**A**) Heatmap analysis of gene expressions in carotenoid pathways. (**B**) Metabolite and enzyme in four processes of carotenoid metabolic pathway. Data are normalized and transformed by log_2_FC of gene expression in zebra-green striped leaves compared to those in zebra-yellow striped leaves (*n* = 3). FC, fold change; Z-G, zebra-green striped in leaves/alternate green leaves; Z-Y, zebra-yellow striped in leaves/alternate yellow leaves.

Importantly, we found that genes encoding zeaxanthin cyclooxygenases (*OsZEP*) of the ABA biosynthetic pathway were enriched in both the carotenoid biosynthesis pathways. Meanwhile, the gene *OsPSY* encoding phytoene synthase (PSY) in the carotenoid biosynthesis pathway was significantly differentially expressed. The two genes involved in metabolic processes such as (E/Z)-phytoene biosynthesis, lutein biosynthesis, and zeaxanthin biosynthesis may be involved in B03S pigment biosynthesis regulation at the transcriptional level (Figure 3B). Importantly, the expression of two DEGs, the ZEP gene *OsZEP* and the phytoene synthase (PSY) gene *OsPSY2*, decreased by 82.41% and 66.28%, respectively, in Z-Y (Figure 4). These results indicate that a substantial proportion of genes are involved in the regulation of secondary metabolism, particularly carotenoid biosynthesis, during the formation of the leaf-color phenotype of B03S resulting from deep transplanting.

Based on these findings, we became interested in the effects of differences in carotenoid synthesis pathways on striped leaves. In order to understand the carotenoid biosynthesis-related pathways at play in the striped leaves of B03S, a heatmap of the expression of twenty-two genes, namely, fourteen genes involved in ABA biosynthesis and eight genes involved in β-carotene biosynthesis, expressed in the striped leaves of B03S was generated (Figure 4). In the Z-Y leaves of B03S, the transcript abundance of the genes involved in β-carotene biosynthesis, the carotenoid pathway, and ABA biosynthesis decreased by 38.99%, 20.80%, and 8.59%, respectively, in the same period. The expression levels of nine genes significantly decreased in the Z-Y leaves. The transcript levels of seven genes were relatively stable, and those of six genes decreased slightly.

### 2.4. Verification of the Expression of Genes Involved in the Carotenoid Pathway in B03S Leaves

Based on the differential expression of certain genes involved in carotenoid biosynthesis in the striped leaves induced by leaf sheath shading, the expression of the genes in the striped leaves of B03S was verified by qPCR to analyze the secondary metabolic network and regulatory mechanism activity induced by deep transplanting, especially the expression of the genes involved in the carotenoid pathway and associated with the xanthophyll cycle. Based on the expression of related genes involved in the carotenoid biosynthesis pathway according to transcriptome analysis, the encoded genes of six key enzymes, namely, the ZEP gene *OsZEP* (LOC_Os04g37960), VDE gene *OsVDE* (LOC_Os04g31040), NCED gene *OsNCDE2* (LOC_Os12g24800), indole-3-acetaldehyde oxidase (AAO) gene *OsAAO* (LOC_Os03g57680), short-chain dehydrogenase/reductase protein-like (SDR) gene *OsSDR* (LOC_Os02g42810), and cytochrome P450 gene *OsP450* (LOC_Os01g43710), were detected in the Z-Y and Z-G stripes of the B03S leaves in response to deep transplanting and in the green CK leaves. In addition, the ABA-responsive gene *OsASR* (LOC_Os12g29400) and the differentially expressed gene Glutamate decarboxylase (*OsGAD*, LOC_Os03g51080) were also selected, and they were detected in the Z-Y, and Z-G stripes of the B03S leaves in response to deep transplanting and in the green CK leaves.

**Figure 5:**
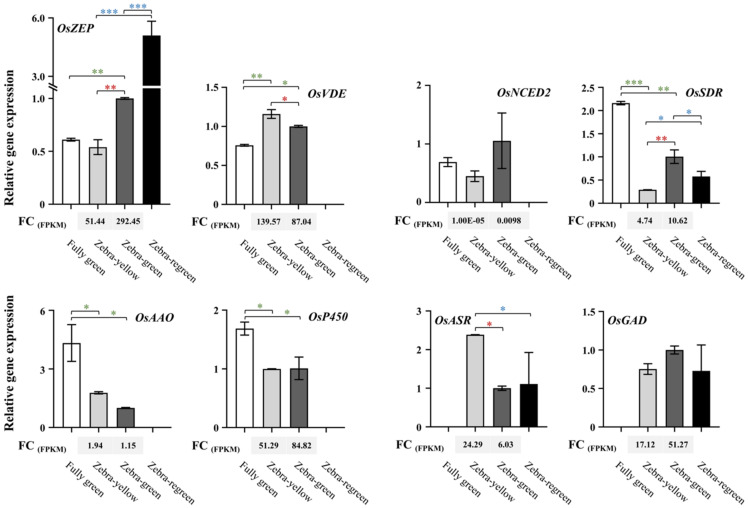
qPCR-based verification analysis of the expression of genes in the striped leaves of B03S. The internal reference gene is *OsActin*. Data are shown as mean ± standard deviation (*n* = 3). Asterisks indicate significant differences revealed by Student’s *t*-test (*, *p* < 0.05; **, *p* < 0.01; ***, *p* < 0.001). Green or blue asterisks indicate the gene expressions of the striped leaves compared to those of full green leaves (CK) or regreen leaves, respectively; Red ones indicate the gene expressions between zebra-green striped and zebra-yellow striped in the striped leaves. FC, fold change; FPKM, fragments per kilobase per million calculated based on transcriptome data.

*OsZEP* is one of the key enzyme-encoding genes involved in response to light stress. Unlike in the normal green leaves from the nontransplanted plants, the expression levels of two genes, *OsZEP* and *OsNCED*, were downregulated, with FCs of 0.88 and 0.65, respectively, in the Z-Y stripes of the B03S leaves, while they were upregulated, with an FCs of 1.64 and 1.53, in the Z-G stripes (Figure 5). In addition, the expression levels of *OsSDR*, *OsAAO*, and *OsP450* were downregulated, with an FC of 0.13–0.59, in both the Z-Y and the Z-G stripes of the striped leaves (Figure 5). In contrast, *OsVDE* expression was upregulated in the striped leaves.

In the striped leaves, with Z-G stripes serving as a control, the transcript abundance of *OsZEP*, *OsP450*, *OsSDR*, *OsNCED,* and *OsGAD* showed a downward trend in Z-Y, and that of *OsVDE, OsAAO*, and *OsASR* was increased in Z-Y, albeit with some differences in gene expression. Notably, in the Z-Y stripes of the B03S leaves, *OsVDE* and *OsAAO* showed higher expression levels (FCs of 1.16 and 1.31, respectively), and *OsZEP* and *OsSDR* were significantly downregulated (FC ranging from 0.28–0.54) (Figure 5). The analysis results showed that the expression of these genes was similar to that revealed by high-throughput sequencing, confirming that the sequencing results and the analysis of the key regulatory metabolites were accurate and reliable.

### 2.5. Analysis of ROS Accumulation in the B03S Leaves according to NBT Staining

**Figure 6:**
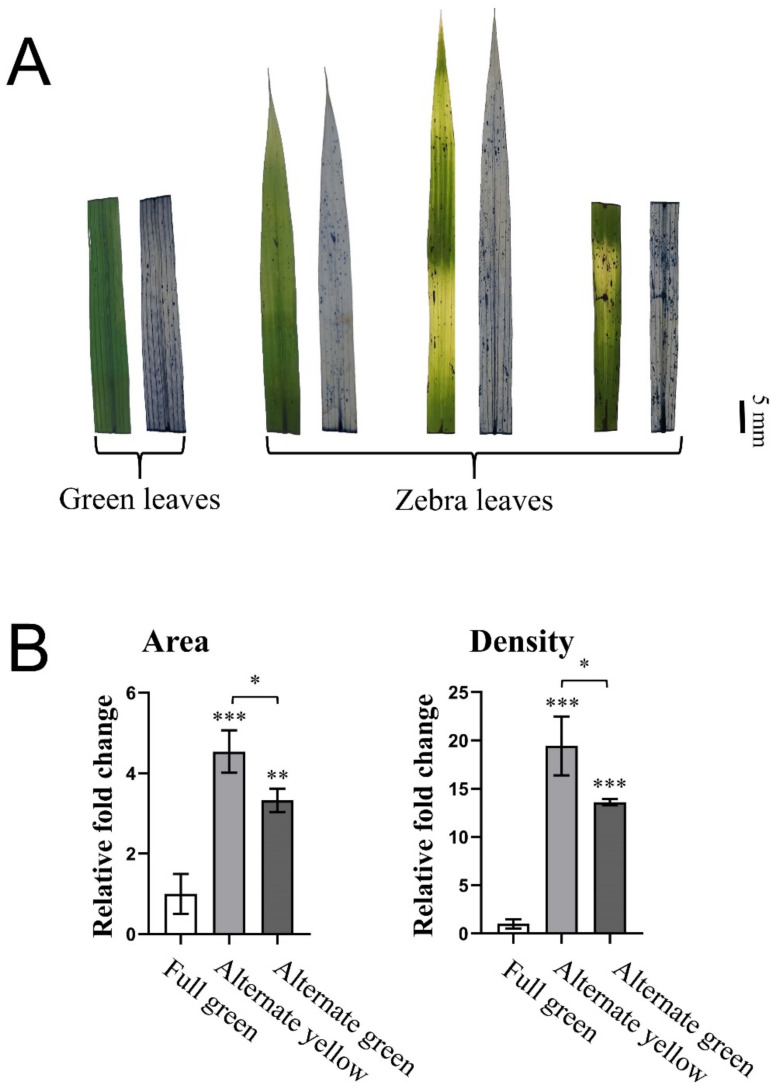
ROS accumulation analysis by NBT staining in the striped leaves of B03S. (**A**) The leaves of B03S by NBT staining (*n* = 3, three individual leaves). (**B**) ROS accumulation analysis of the staining leaves. Data are shown as mean ± standard deviation (*n* = 3, three regions with the radius of 5 mm of the individual leaves). Asterisks indicate significant differences revealed by Student’s *t*-test (*, *p* < 0.05; **, *p* < 0.01; ***, *p* < 0.001).

The typical pigment metabolites identified as carotenoids, which are generally acknowledged to have a certain potential antioxidant capacity, were assessed on the basis of their ability to quench ROS via NBT staining of the striped leaves. The results showed that the density of ROS was significantly higher in the striped leaves than in the fully green leaves (Figure 6). In the striped leaves, the ROS density was higher in the Z-Y stripes than in the Z-G stripes (Figure 6A). By quantitative analysis of the ROS, the distribution areas in the Z-Y and Z-G stripes of the striped leaves were 4.54-fold and 3.33-fold greater, respectively, than were those of the fully green leaves, and the average density in the striped leaves was 13.61–19.44-fold higher than that in the fully green leaves (Figure 6B).

## 3. Discussion

### 3.1. Dark Shading of the Leaf Sheath Induces Albinism in the Leaves of B03S

In this study, B03S is a PTGMS rice line that is affected by the photoperiod but exhibits mainly temperature-sensitive characteristics and has a critical temperature for male sterility [27]. The characteristic leaf-color phenotype of B03S is an albino trait that results in striped leaves displaying yellow-green intermittent stripes. The cause of these striped leaves was explored in our previous studies. The preliminary results pointed to deep transplanting rather than root injury for the induction of B03S traits under transplantation, and root reduction and hydroponic treatments were imposed. Furthermore, the transplanting depth was also determined to be 4–5 cm when gradients ranging from 0 cm to 5 cm were evaluated (data unpublished). In this study, the direct cause of the B03S striped leaves was confirmed to be hiding the leaf sheath in the dark, as additional treatments involving soil covering aluminum foil shading through pot experiments were imposed (Figure 1, Table 1). Furthermore, the striped-leaf traits of B03S occurred when the critical shading range of the plant reached or exceeded 4 cm above the base of the leaf sheath. In addition to the seedling stage, the zebra stripes of B03S occurred after deep transplanting and were mostly distributed in the new leaves and the leaf sheaths at any time throughout the whole period of rice planting. At the same time, the striped-leaf traits were maintained for two or three new leaves for approximately 15 days (Figure 1C). Above all, the color of these leaves subsequently returned to normal green, and the agronomic traits of plants were not subsequently affected.

Light and temperature have an important impact on traits [28]. The maize albino mutant *eal1* has some sensitivity to temperature, and the seedlings have a higher pigment content and a faster leaf color recovery rate at high temperatures [29]. The yellow leaf tomato mutant *ym* exhibited pigment deprivation and accumulated substantial ROS under dark treatment [30]. Leaf color expression of rice *2177S* mutant produced after physical mutation is related to temperature, with *W4* and *W11* bleaching at 30–35 °C, in contrast, *W17* and *W25* bleaching at 15–25 °C, and leaf color regreening with increasing temperature [31]. In addition, the degree and retention of the leaf color were affected by temperature. Under preferred temperatures (23 °C to 28 °C), striped leaves were typical for B03S, with three or more striped leaves whose patterns are maintained for a long time. At low temperatures (below 21 °C) or high temperatures (above 32 °C), the zebra stripes are atypical or insignificant, with only 1–3 striped leaves whose patterns persist for a short time (data unpublished).

Previously, many studies and reviews have shown that transplanting or root injury is a necessary expression condition for zebra leaf traits, and it is believed that root damage caused by transplanting will lead to hormones such as ABA, which will affect zebra leaf production. Apart from hormone signaling, we found that sugar signaling affects leaf stripe and chloroplast development, which will be the focus of our next research.

### 3.2. Typical Carotenoids That Enter the Xanthophyll Cycle Are Involved in the Formation or Regulation of Albinism in Leaves

For the PTGMS line B03S, the striped-leaf trait is a result of the formation of interruptions of albino leaf sheaths and leaves, which results in an extraordinary leaf-color phenotype. The striped-leaf trait is closely related to photosynthetic pigments and chloroplast growth and development. Previous studies showed a significant decrease in the level of chlorophyll, abnormal development of chloroplast structure, and a decreased overall photosynthetic capacity in striped leaves of B03S, and the changing trend was consistent with the bleached parts of the striped leaves (data unpublished). In addition, the activities of peroxidase and superoxide dismutase were increased in the striped leaves.

**Figure 7:**
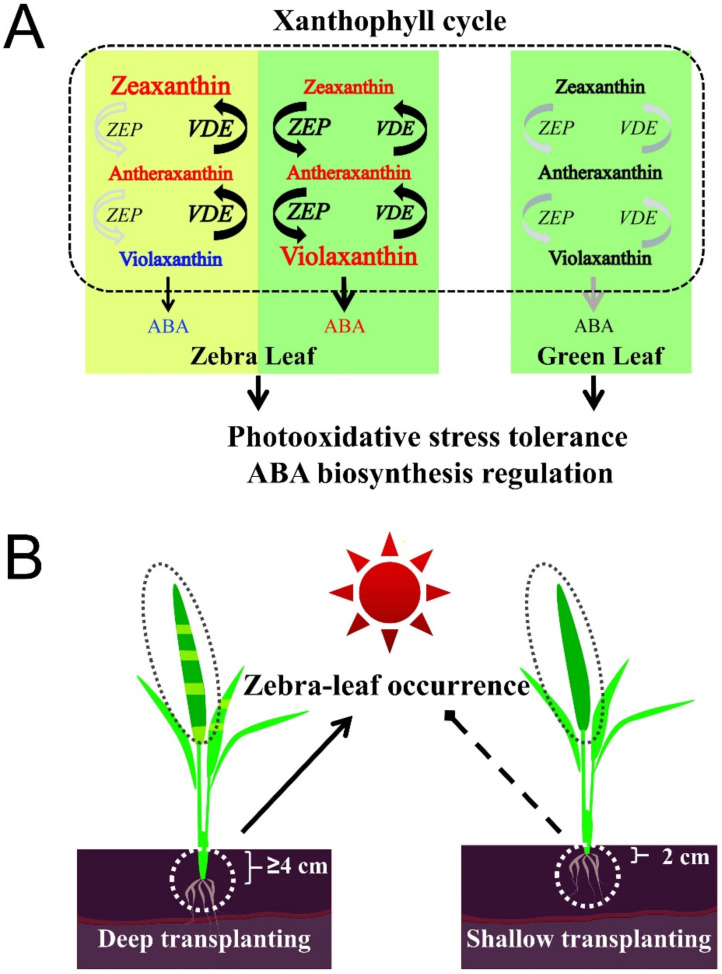
Patterns of zebra leaf occurrence and metabolic regulation. (**A**) Work pattern of xanthophyll cycle in regulation mechanism in rice zebra leaf. (**B**) Occurrence pattern of zebra leaf in rice. Metabolites downregulated/upregulated in rice leaves are marked in blue/red color according to our results of B03S. Gene expressions of xanthophyll cycle upregulated in leaves are marked with the black and increased font according to data in the study.

The abnormal light environment of plant growth causes plant adaptation through a range of physiological and metabolic levels. Previous studies have shown that the pigment content in Z-Y was significantly reduced compared to Z-G, with carotenoids decreasing by nearly 67.7% (data unpublished). Light can significantly affect carotenoid content, especially the xanthophyll cycle components. This physiological change contributes to enhanced photosynthesis, carbon assimilation, non-photochemical quenching, and reactive oxygen species clearance [32,33,34]. This study focuses on the identification of pigment metabolites in the yellow-green stripes of B03S striped leaves. In the striped leaves, the overall metabolism of carotenoids increased (Figure 2), particularly that of octahydrolycopene, an important intermediate metabolite upstream of the carotenoid metabolic pathway; this increase was found by the synchronous increases in both the green and the yellow stripes of the striped leaves (Figure 2). Remarkable distinctions in the metabolic flow of the carotenoid pathway components can be drawn between the green and yellow stripes of the striped leaves. In the Z-Y leaves, the differentially accumulated metabolites were enriched in the lutein biosynthesis pathway, with zeaxanthin being the most typical representative metabolite identified (Figure 2 and Figure 7). Among the differentially accumulated carotenoids, zeaxanthin largely accumulated; this metabolite is synthesized via the oxidation of antheraxanthin by a key enzyme ZEP rather than via entry into the downstream ABA biosynthesis pathway or lutein synthesis pathway for photoprotective carotenoids. However, the contents of most carotenoids and their derivatives, such as the main metabolites in the α-carotene, β-carotene, δ-carotene, cryptoxanthin, lutein, zeaxanthin, pyrrhoxanthin, neoxanthin, and other metabolites, were increased in Z-G leaves (Figure 2). The three major compounds in the xanthophyll cycle are violaxanthin, flaxanthin, and zeaxanthin [35]. The degree of lutein de-epoxidation indicated by the ratio of the three compounds in striped leaves was generally higher than that in the normal green leaves, which is also higher than that in Z-G leaves (Table 2). Accordingly, the thermal dissipation capacity for B03S leaves reflected by the xanthophyll cycle in descending order is as follows: Z-Y leaves, Z-G leaves, and CK leaves.

In the carotenoid metabolic pathway, intermediate metabolites are generally recognized to play a role in oxidation resistance or alleviating adverse effects in plants under abiotic stress [36]. Most carotenoids, such as zeaxanthin, antheraxanthin, and neoxanthin, are antioxidant-type pigments, which previous studies have reported play a certain role in dissipating excess light energy in photosynthetic organs. In addition, ABA is the key plant stress hormone that accumulates and enhances the ability of plants to resist unfavorable environmental conditions when plants experience stress [37,38,39]. The carotenoid neoxanthin is the direct precursor of ABA and is synthesized downstream of the carotenoid metabolic pathway. Studies have shown that 200 mg/L of exogenous ABA was applied to affect petal pigment accumulation and deepen petal color by increasing the total carotenoid content, indicating that ABA can also affect the formation of plant tissue color [40]. From the perspective of ABA metabolism, the synthesis of ABA is blocked under light, and a large amount of ROS produced in striped leaves fails to be removed in time, resulting in damage to the developing chloroplast structure. In turn, chlorophyll cannot be normally supplied to the leaves, resulting in the zebra-stripe characteristics during leaf greening. ABA plays an important role in plant growth and development and stress adaptation and also has an impact on leaf color mutations, with overexpression of rice NAC transcription factor ONAC054 under dark and ABA treatment [41]. ABA is a key regulator of anthocyanin synthesis during the tea leaf color transition, and the related genes involved in ABA signaling were significantly and positively correlated with the photoperiod, indicating that illumination plays an important role for ABA in regulating leaf color [42,43].

### 3.3. Regulatory Zeaxanthin Accumulates via Increased OsZEP and OsVDE Expression in Leaves to Alleviate Photooxidative Stress

Carotenoids play multiple roles in the plant growth and development process. As photoresponsive pigments of the photosynthetic system, carotenoids are also important signals of oxidative stress and ROS [44]; moreover, carotenoids can protect photosynthetic elements from ROS under adverse conditions [45]. The xanthophyll cycle dissipates excess light energy mainly through thermal dissipation inhibitory mechanisms and the photochemical quenching mechanism, which are important mechanisms to protect the plant photosynthetic system [46]. The xanthophyll cycle was found to be more active in the striped leaves than in the normal leaves; that is, it is effective at protecting photosynthetic organs and enhancing the adaptability of B03S during abnormal development or under stress conditions (Figure 7). The large reduction in the abundance of protective carotenoids in the Z-Y leaves prevents photooxidative stress from being effectively alleviated, which may lead to the low efficiency of removing ROS and the failure to remove excess ROS in time, resulting in photoinhibition and photobleaching and then albinism phenomenon (Figure 7). When largely abundant, zeaxanthin can dissipate excess light energy and protect photosynthetic organs from photooxidative stress [47].

At the molecular regulatory level, excess light energy and acidification of the thylakoid cavity activate VDE, and deoxygenation then leads to violaxanthin and zeaxanthin synthesis [48]. In the xanthophyll cycle, the three major lutein species of violaxanthin, flaxanthin, and zeaxanthin, and extensive accumulation of zeaxanthin can best remove excess ROS from the leaves [49]. Moreover, zeaxanthin plays a role in the two photoprotective defense mechanisms, namely, thermal dissipation and ROS removal, to protect against light stress and ensure normal plant growth [50]. The abundance of zeaxanthin in the striped leaves constitutes an adaptive protective mechanism for rice to alleviate the adverse effects of surrounding albino leaf stripes on plant growth and development.

Studies have shown that there is a direct C15 pathway and a C40 indirect pathway for ABA biosynthesis. However, the main source of ABA is the C40 indirect pathway, through which ABA is indirectly synthesized by oxidative cleavage of the carotenoid metabolites. In this pathway, ZEP and NCED play key roles [51]. ZEP functions in the xanthophyll cycle through reversible reactions regulating VDE, which results in the conversion of zeaxanthin to neoxanthin, determining the reaction direction of the xanthophyll cycle and the metabolic direction of the downstream ABA pathway [52,53]. NCED functions downstream of the whole carotenoid metabolic pathway, and NCED activity represents the key step in the regulation of the C40 indirect synthesis pathway: 9-cis-flavin is converted into flavanol aldehyde, the process of which constitutes the key rate-limiting enzyme of ABA synthesis in higher plants, and NCED expression is closely related to the amount of ABA synthesis [54]. When the transcriptome data and qPCR data were combined, it was found that expression of the *OsZEP* and *OsNCED* genes was significantly downregulated, while that of *OsVDE* was upregulated; the metabolites flowed in the opposite direction of the xanthophyll cycle, and the ABA synthesis pathway was also inhibited in the Z-Y leaves (Figure 7). The expression of these genes was regulated in Z-G leaves, with significantly upregulated expression of *OsZEP*, *OsVDE*, and *OsNCED* (Figure 5). Compared with normal green leaves, the green stripes of striped leaves presented more active carotenoid metabolic pathways, especially the xanthophyll cycle and ABA synthesis (Figure 7).

During striped leaf formation, the carotenoid metabolic pathway actively changed, which reflected a positive adaptation to the adverse effects caused by chlorophyll deficiency. The xanthophyll cycle plays a key role in physiological and metabolic regulation. This role is in addition to the response of this pathway to light stress through the dissipation of excess light energy. Other roles included processes that involve enzymes (ZEP, VDE) [55]; changes in the control and direction of metabolic pathways, which affects the downstream synthesis of the stress hormone ABA; enrichment and use of cytosolic oxidative damage; protection of the integrity of the cell membrane and leaf growth and development; reduction in photosynthetic organ damage; and improvements to the tolerance of abnormal light conditions, ultimately aiming to maintain plant biological activities at levels as normal as possible. Mutants of *ZEP* and *VDE* have been found in various plants, including Arabidopsis *npq1*, *npq2*, tomato *hp3*, and microalgae *lhcx1* [56,57,58]. The downregulation of *ZEP* is often accompanied by excessive accumulation of zeaxanthin and shows reduced levels of downstream metabolites such as ABA [59].

## 4. Materials and Methods

### 4.1. Plant Material and the Leaf Color Phenotype

A natural leaf color mutant from rice (*Oryza sativa* L. ssp. *indica*) cultivar Efeng 1S, a PTGMS rice line, was crossed with conventional rice (*Oryza sativa* L. ssp. *indica*) cultivar 9311 for six generations by our research team. The resulting mutant exhibited stable male fertility, critical temperature thresholds, and extraordinary leaf color stripe patterns. The leaf sheaths and leaves of B03S had albino stripes interrupting green ones, ultimately appearing as yellow-green intermittent stripes that resemble zebra stripes. In addition to occurring at the seedling stage, these zebra stripes can occur after deep transplanting throughout almost the entire vegetative growth phase, mostly concentrated within new leaves and/or sheaths. These striped-leaf traits occur on two or three new leaves and persist for approximately 12–15 days. These leaves then gradually return to a full, normal green color.

### 4.2. Phenotypic Analysis by Experimental Treatments

Pot and field experiments were performed at the Crop Physiology and Production Centre of Huazhong Agricultural University in Wuhan (30°47′ N, 114°35′ E) under natural conditions from May to October in 2020 and 2021. B03S was sown into pots 40 cm wide, 60 cm long, and 20 cm tall and allowed to grow. From 30 days (3-leaf stage) to 38 days (5-leaf stage) after planting, four treatments involving the base leaf sheath were imposed to induce striped leaves: (1) deep/shallow transplanting, in which plants in pots and in the field were transplanted such that 4 cm/2 cm of the plant was below ground, respectively; (2) soil covering, in which plants in pots were covered with soil to a height of 4 cm; (3) aluminum shading, in which plants in pots were covered with aluminum foil to a height of 4 cm; and (4) a control, in which seeds were sown (not transplanted) in pots and in the field. The plants were subsequently grown under natural conditions and managed in accordance with regular fertilization, watering, and disease management practices.

Because in situ shading (soil covering and aluminum shading) makes it difficult to control a single condition in the field, it was applied only to the potted plants for trait observation and confirmation of the cause of the trait. Due to the differences in soil fertility, the nutrient availability in the field and in the pots is obviously different, and the seedling traits in the field were more typical, so samples were collected from the materials in the field. The distribution of ROS was determined by nitroblue tetrazolium (NBT) in fresh samples from deep/shallow transplanted seedlings. Samples were taken routinely during the typical and stable period (1–2 weeks after transplanting). Striped leaves/all-green leaves of plants that were deep/shallow transplanted were collected. Striped leaf material was sampled routinely according to the dynamic changes in the striped leaves. Transcriptome sequencing was performed on the leaves of deep/shallow transplanted seedlings, and the yellow (Z-Y) and green (Z-G) leaf parts were collected during the stable period of the striped-leaf trait. Targeted metabolome sequencing was performed on the leaves (Z-Y)/(Z-G) of deeply transplanted seedlings and on the green leaves (CK) of shallow transplanted seedlings during typical and stable periods (7 days after transplanting). The leaves were sampled in the field, quickly immersed in liquid nitrogen, and placed in a centrifuge tube in a −80 °C cryogenic refrigerator for subsequent experiments.

### 4.3. Ultra-Performance Liquid Chromatography/Tandem Mass Spectrometry (UPLC–MS/MS) Analysis of Leaf Color Metabolites Composing Carotenoids

Separate yellow and green parts of the leaves of B03S seedlings in the deep transplanting group and the fully green leaves of the control (CK) group of nontransplanted plants were subjected to targeted metabolomics analysis. There were three biological replicates per treatment. The metabolomes were determined by Metware (Wu Han, China) and performed in reference to existing methods [60].

Freeze-dried samples were ground to a fine powder in a ball mill (MM 400, Retsch) with zirconia beads at 30 Hz. The powder was extracted with 0.5 mL of an n-hexane, acetone, and ethanol solution consisting of 0.01% (*m/v*) butylated hydroxytoluene (BHT) and then dissolved in a 100 μL solution of methanol and methyl tert butyl ether by vortexing. Before UPLC-MS/MS analysis, the extracts were centrifuged at 12,000 rpm for 10 min and then filtered (SCAA-104, 0.22 μm pore size; ANPEL, Shanghai, China; http://www.anpel.com.cn/ (accessed on 1 March 2022)). The samples were analyzed using a UPLC-APCI-MS/MS system(SCIEX, Framingham, MA, US) (UPLC ExionLC™ AD, https://sciex.com.cn/ (accessed on 1 March 2022); MS Applied Biosystems 6500 Triple Quadrupole, https://sciex.com.cn/ (accessed on 1 March 2022)). Linear ion trap and triple quadrupole scans were acquired on a triple quadrupole-linear ion trap mass spectrometer and controlled by Analyst 1.6.3 software (AB-SCIEX, Framingham, MA, USA). The carotenoid contents were detected by MetWare (http://www.metware.cn/ (accessed on 1 March 2022)). The determination of the metabolome involved the use of a carotenoid standard solution as an internal reference.

### 4.4. RNA Extraction, Sequencing and Expression Analysis in B03S Leaves

Total RNA was extracted from the yellow and green parts of the B03S leaves using RNAiso Plus Total RNA Extraction Reagent (Takara, Beijing, China) following the manufacturer’s instructions, and the RNA integrity was checked via an Agilent Bioanalyzer 2100 (Agilent Technologies, Santa Clara, CA, USA). The qualified total RNA was further purified by an RNeasy Micro Kit (QIAGEN, GmBH, Hamburg, Germany) in conjunction with RNase-Free DNase (QIAGEN, GmBH, Hamburg, Germany).

RNA sequencing and analysis were conducted by staff at SHBIO (Biotechnology, Shanghai, China) and via its online platform (http://www.shbio.com (accessed on 1 May 2022)). The mRNA library was prepared from 1 μg of total RNA via a TruSeq sRNA Kit (Illumina, San Diego, CA, USA) and a cBot Clonal Amplification System (Illumina, San Diego, CA, USA). mRNA sequencing was performed on an Illumina HiSeq 2500 (Illumina, San Diego, CA, USA), with three biological replicates.

#### 4.4.1. Quality Control

Raw data (raw reads) of fastq format were firstly processed through in-house perl scripts. In this step, clean data (clean reads) were obtained by removing reads containing adapter, reads containing ploy-N, and low-quality reads from raw data. At the same time, Q20, Q30, and GC content of the clean data were calculated. All the downstream analyses were based on clean data with high quality.

#### 4.4.2. Reads Mapping to the Reference Genome

Reference genome and gene model annotation files were downloaded from genome website directly. Index of the reference genome was built using Hisat2 v2.0.5 and paired-end clean reads were aligned to the reference genome using Hisat2 v2.0.5. We selected Hisat2 as the mapping tool for that Hisat2 can generate a database of splice junctions based on the gene model annotation file and thus a better mapping result than other non-splice mapping tools.

#### 4.4.3. Quantification of Gene Expression Level

Counts v1.5.0-p3 was used to count the reads numbers mapped to each gene, and then FPKM of each gene was calculated based on the length of the gene and reads count mapped to this gene. FPKM, expected number of Fragments Per Kilobase of transcript sequence per millions base pairs sequenced, considers the effect of sequencing depth and gene length for the reads count at the same time, and is currently the most commonly used method for estimating gene expression levels.

#### 4.4.4. Differential Expression Analysis

Differential expression analysis of two conditions/groups (two biological replicates per condition) was performed using the DESeq2 R package (1.20.0). DESeq2 provides statistical routines for determining differential expression in digital gene expression data using a model based on the negative binomial distribution. The resulting *p*-values were adjusted using Benjamini and Hochberg’s approach for controlling the false discovery rate. Genes with an adjusted *p*-value ≤ 0.05 found by DESeq2 were assigned as differentially expressed. Prior to differential gene expression analysis, for each sequenced library, the read counts were adjusted by edgeR program package through one scaling normalized factor. Differential expression analysis of two conditions was performed using the edgeR package (3.22.5). The *p*-values were adjusted using the Benjamini and Hochberg method. Corrected *p*-value of 0.05 and absolute foldchange of 2 were set as the threshold for significantly differential expression.

#### 4.4.5. GO and KEGG Enrichment Analysis of Differentially Expressed Genes

Gene Ontology (GO) enrichment analysis of differentially expressed genes was implemented by the cluster Profiler R package, in which gene length bias was corrected. GO terms with corrected *p*-value less than 0.05 were considered significantly enriched by differential expressed genes. KEGG is a database resource for understanding high-level functions and utilities of the biological system, such as the cell, the organism, and the ecosystem, from molecular-level information, especially large-scale molecular datasets generated by genome sequencing and other high-throughput experimental technologies (http://www.genome.jp/kegg/ (accessed on 24 November 2021)). We used cluster Profiler R package to test the statistical enrichment of differential expression genes in KEGG pathways.

### 4.5. Real-Time Quantitative PCR (qPCR)-Based Validation of Gene Expression

The qPCR was performed to analyze the relative gene expression of *OsZEP*, *OsVDE*, *OsNCED2*, *OsAAO*, *OsSDR*, *OsP450, OsASR,* and *OsGAD* in the yellow and green parts of the B03S leaves compared with the fully green leaves (CK). The primers of the six genes were designed by the use of the NCBI database and synthesized by Bioengineering Co., Ltd. (Shanghai, China; Table 3), with *OsActin* (GenBank Accession, X16820.1) serving as a reference gene. Total RNA was extracted using an RNAprep Pure Plant Kit (TIANGEN, Beijing, China) as described by the manufacturer. For qPCR-based verification of gene expression, 3.0 µg of RNA from each sample was used, with three biological replication included. First-strand cDNA was synthesized using a ReverTra Kit (Toyobo, Osaka, Japan). qPCR was then performed using SYBR Green SuperMix (Bio-Rad, Hercules, CA, USA) on a QuantStudio 6 Flex instrument (Applied Biosystems, Foster, CA, USA). The relative expression was analyzed according to the 2^−ΔΔCT^ method [61,62]. The expression levels were normalized against the expression level of the *Actin7* gene [63].

### 4.6. Histochemical Staining of ROS in B03S Leaves

For analysis of ROS accumulation, the O_2_^−^ in the B03S leaves was stained with NBT (Yuanye, Shanghai, China) according to the methods of Kumar [64,65]. First, the leaves were cut into 10 cm long samples and immersed in phosphate buffer (pH 7.5) consisting of 2 mM NBT, 42 mM Na_2_HPO_4_ and 8 mM NaH_2_PO_4_, followed by vacuum infiltration at room temperature for 12 h. The NBT and O_2_^−^ combined, forming visible blue stains in the leaves. Then, the leaves were decolorized with 100% ethanol for 1 h. Finally, the decolorized leaves were soaked in glycerol and imaged with a digital camera (EOS RP, Canon, Japan) under visible light.

### 4.7. Data Analysis

The values were subjected to analysis of variance (ANOVA), and significant differences (*, *p* < 0.05; **, *p* < 0.01; ***, *p* < 0.001) were analyzed using Excel software (Microsoft Corp., Redmond, WA, USA). Images were combined with PowerPoint software (Microsoft Corp., Redmond, WA, USA) and Photoshop software (Adobe, San Jose, CA, USA). Metabolites whose abundance presented a fold-change (FC) ≥ 1.7 or an FC ≤ 0.6 and *p*-value ≤ 0.05 were considered distinctly differentially accumulated compounds between two samples [66]. DEGs of transcriptome whose abundance presented a fold-change (FC) ≥ 2.0 or an FC ≤ 0.5 and *p*-value ≤ 0.05 were considered distinctly differentially accumulated between two samples. ROS accumulation analysis of the staining leaves with more than three regions of the 5 mm radius from individual leaves.

## 5. Conclusions

In conclusion, we have further confirmed the previous inference about the striped leaf pattern induced in response to transplanting in situ and in response to different shading methods, and we propose that light/darkness is a direct environmental factor leading to this leaf-color phenotype. The sensitive site is the leaf sheath. Regarding the metabolic and physiological aspects, the carotenoid metabolic pathway undergoes active changes during the formation of striped leaves, with more metabolites entering the xanthophyll cycle, which specifically significantly increased the zeaxanthin content in the albino stripes of the striped leaves through the regulatory expression of the *OsZEP*-*OsVDE* gene pair, thus removing excess ROS in the leaves.

Taken together, zeaxanthin biosynthesis, *OsZEP,* and *OsVDE* regulate rice-striped leaves occurring in response to deep transplanting. The results provide new insight for further analysis of the mechanism of uneven leaf color distribution in nature.

## Figures and Tables

**Table 1:** Phenotype analysis of zebra leaves occurred under different treatments.

Groups	Root Damage	Shading Treatment	Shading Region	Leaf-Colored Characteristic
Shallow Transplanting	Yes	Yes	2 cm	No
Deep Transplanting	Yes	Yes	4 cm	Yes
Soil Covering	No	Yes	4 cm	Yes
Aluminum foil Covering	No	Yes	4 cm	Yes
Nontransplanting	No	No	0 cm	No

Yes/No indicate significant differences in leaf-colored characteristic under different treatments (*n* = 120).

**Table 2:** Deepoxidation analysis of xanthophyll cycle in zebra leaves.

Metabolite	Fully Green Leaf	Alternate Yellow Leaf		Alternate Green Leaf	
Z (μg/g)	9.43 ± 7.56	43.44 ± 8.31	**	25.17 ± 3.19	
A (μg/g)	27.65 ± 11.99	48.88 ± 1.98	*	52.46 ± 4.36	*
V (μg/g)	80.47 ± 11.98	63.59 ± 1.07		111.20 ± 3.36	**
Ratio (%)	31.54 ± 6.27	59.21 ± 1.95	***	41.11 ± 1.22	

Data are shown as mean ± standard deviation (*n* = 3). Asterisks indicate significant differences revealed by Student’s *t*-test (*, *p* < 0.05; **, *p* < 0.01; ***, *p* < 0.001). Z, zeaxanthin; A, anther antheraxanthin; V, violaxanthin; Ratio, (Z + A)/(Z + A + V) ∗ 100%.

**Table 3:** Primer pair information of candidate genes.

Gene Name	Gene ID	Forward Primer Sequence (5′–3′)	Reverse Primer Sequence (5′–3′)
*OsZEP*	LOC_Os04g37619	TCTAGGAGGAAACAGCACGA	CCAATGCATCGTCATCCTC
*OsVDE*	LOC_Os04g31040	CTCTAAGCAGCATCA	GCCAGCTCTATTCTGCACT
*OsNCED2*	LOC_Os12g24800	TTGGTGTAAGTGGTC	ACCCTAGACCAAAGTCACGA
*OsAAO*	LOC_Os03g57680	TTGTTGATTCCTCGC	ATACACTGCCTCCCCAGAA
*OsSDR*	LOC_Os03g59610	CACAAACTACTTGG	ACGGTTCTACGCACATCTT
*OsP450*	LOC_Os01g43710	CTGAAAGAGCACGGGAAACT	CATACGTTACAACTCCGCC
*OsASR*	LOC_Os12g29400	TCGGATGGAACGATGAGC	CAATAGCGACCTGGACAAACT
*OsGAD*	LOC_Os03g51080	GCTGAAACAGGCTGGGACA	GTTGAGGGTGAAGGTGGG

## Data Availability

Not applicable.

## Supplementary Materials

The following supporting information can be downloaded at: https://www.mdpi.com/article/10.3390/ijms23158340/s1.

## Abbreviations

AAO, Abscisic Aldehyde Oxidase; ABA, Abscisic Acid; DEGs, Differentially Expressed Genes; FDR, False Discovery Rate; LC-MS, Liquid Chromatograph Mass Spectrometer; NBT, Nitrotetrazolium Blue Chloride; NCED, 9-Cis-Epoxycarotenoid Dioxygenase; PCR, Polymerase Chain Reaction; PSY, Phytoene Synthase; qRT-PCR, Quantitative Real Time-PCR; ROS, Reactive Oxygen Species; VDE, Violaxanthin De-epoxidase; ZEP, Zeaxanthin Epoxidase.
