# Supplementary material for: Cia Zeaxanthin Biosynthesis, OsZEP and OsVDE Regulate Striped Leaves Occurring in Response to Deep Transplanting of Rice
Source: Int J Mol Sci. 2022 Jul 28;23(15):8340. doi: 10.3390/ijms23158340 (PMC9369140; doi:10.3390/ijms23158340)
Supplement: Supplementary file 1 [file ijms-23-08340-s001.zip › ijms-1796953-supplementary.pdf]

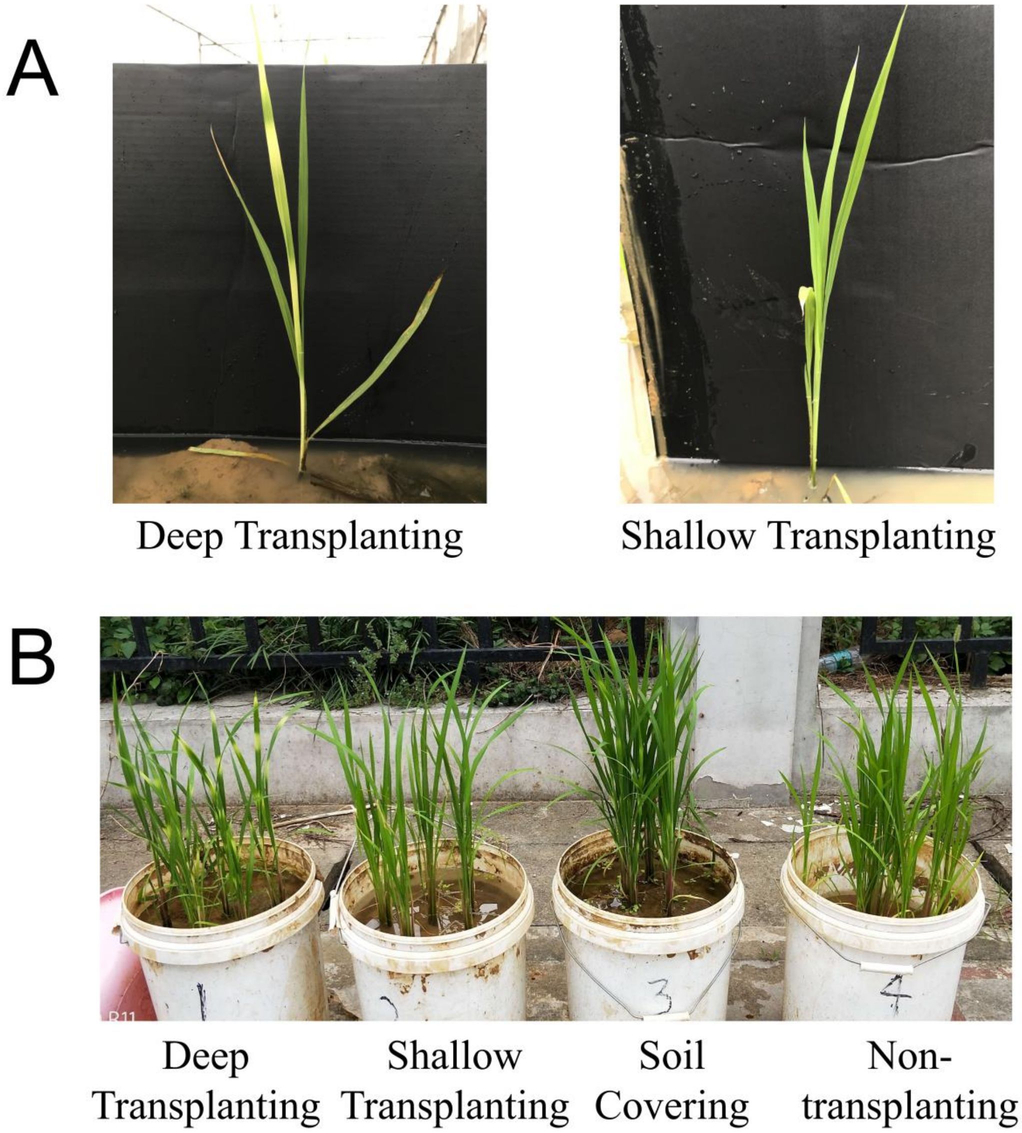

Supplementary Figure S1. Zebra-stripes leaves occurred to varying degrees in B03S under different treatments. **A**. Deep/shallow transplanting in the field. **B**. Deep/shallow transplanting, soil covering and CK plants in pots.

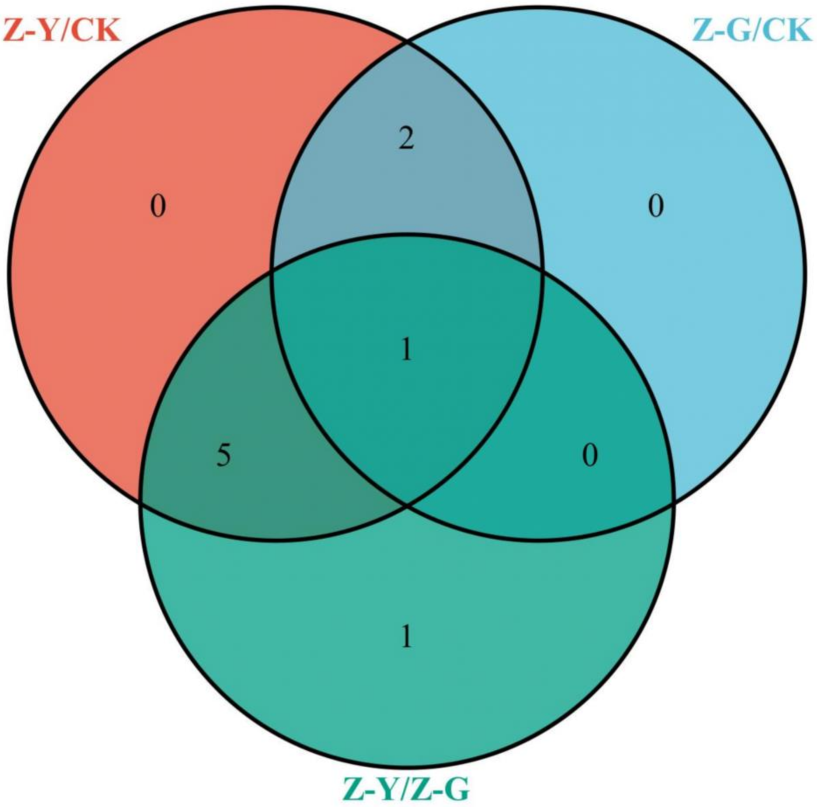

Supplementary Figure S2. Wayne diagram analysis of the differentially accumulated metabolites in the leaves of B03S. CK, full green leaves; Z-G, zebra-green striped in leaves/alternate green leaves; Z-Y, zebra-yellow striped in leaves/alternate yellow leaves.

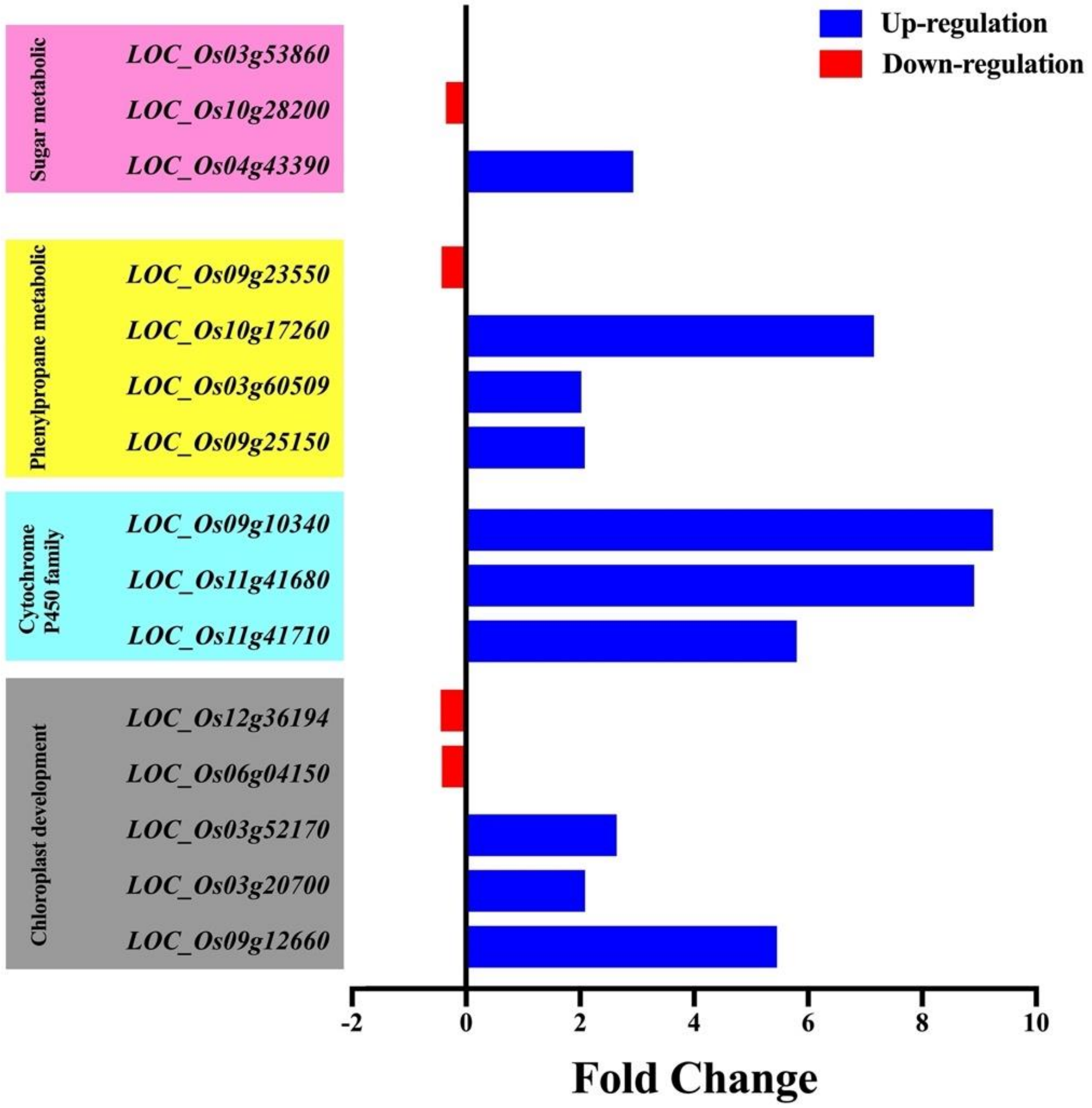

Supplementary Figure S3. Differential expression fold change of DEGs in different regulatory pathways between the Z-G and Z-Y comparison groups. In the figure, blue indicates upregulated expression in Z-G compared to Z-Y; red indicates downregulated expression. Z-G, zebra-green striped in leaves/alternate green leaves; Z-Y, zebra-yellow striped in leaves/alternate yellow leaves.

Supplementary Table S1. KEGG analysis of the differentially expressed genes in the leaves of B03S based on transcriptome sequencing.

KEGG	Pathway	Gene ID	Description	Regulation	Fold change	*P*-value
ko00195	Photosynthesis	LOC_Os04g16872	\	Up	2.54	3.50E-10
		LOC_Os10g21212	\	Up	2.15	1.72E-03
		LOC_Os04g16874	\	Up	2.13	3.54E-04
		LOC_Os10g21192	\	Up	3.37	1.10E-121
		LOC_Os10g39880	Photosystem Q	Up	3.43	2.76E-31
		LOC_Os04g16770	\	Up	3.52	5.74E-98
		LOC_Os08g35420	\	Up	3.32	7.74E-40
		LOC_Os10g41689	Photosystem II D2 protein	Up	3.18	9.87E-04
		LOC_Os10g38234	\	Up	2.36	1.99E-03
		LOC_Os07g05480	\	Down	0.47	2.67E-113
		LOC_Os07g38000	\	Down	0.35	3.85E-08
		LOC_Os04g33630	\	Down	0.32	1.96E-11
ko00830	Retinol metabolism	LOC_Os11g10520	Alcohol dehydrogenase 2	Up	7.47	4.39E-12
		LOC_Os05g34770	Cytochrome c	Up	2.13	3.04E-09
		LOC_Os11g07930	Short-chain dehydrogenase Tic32	Up	2.86	1.45E-04
ko00053	Ascorbate and aldarate metabolism	LOC_Os07g49400	L-ascorbate peroxidase 2	Up	2.15	1.43E-33
		LOC_Os12g08810	VTC2	Up	7.90	1.21E-14
		LOC_Os12g29760	Oxidoreductase, aldo/keto reductase family protein	Down	0.45	6.97E-06
		LOC_Os10g28200	GDP-mannose 3,5-epimerase 1	Down	0.35	3.24E-19
ko00980	Metabolism of xenobiotics by cytochrome P450	LOC_Os11g10520	Alcohol dehydrogenase 2	Up	7.47	4.39E-12
		LOC_Os05g34770	Cytochrome c	Up	2.13	3.04E-09
		LOC_Os04g44924	\	Up	3.86	6.45E-08
		LOC_Os07g07320	\	Up	3.50	6.06E-15

		LOC_Os05g36290	\	Up	2.67	1.87E-03
		LOC_Os10g38710	Glutathione transferase	Down	0.27	2.30E-71
ko00196	Photosynthesis - antenna proteins	LOC_Os03g39610	Chlorophyll a-b binding protein	Up	2.11	3.34E-44
		LOC_Os05g34770	Cytochrome c	Up	2.13	3.04E-09
		LOC_Os08g39140	Heat shock protein 81-1	Up	3.57	3.19E-10
		LOC_Os09g26810	\	Down	0.37	7.61E-43
ko00860	Porphyrin and chlorophyll metabolism	LOC_Os03g20700	Magnesium-chelatase subunit ChlH	Up	2.09	5.08E-05
		LOC_Os04g52130	Oxygen-dependent coproporphyrinogen-III oxidase	Down	0.46	5.01E-22
		LOC_Os06g04150	\	Down	0.41	4.04E-02
ko00740	Riboflavin metabolism	LOC_Os12g35580	Riboflavin synthase, alpha subunit family protein	Down	0.43	1.24E-06
ko04146	Peroxisome	LOC_Os03g38730	Peroxisomal membrane protein	Up	2.67	2.40E-08
		LOC_Os09g14600	\	Up	2.00	6.91E-04
		LOC_Os05g04170	\	Up	2.87	9.92E-04
		LOC_Os03g22810	Superoxide dismutase [Cu-Zn] 1	Up	2.13	6.20E-11
		LOC_Os12g35890	Sarcosine oxidase	Down	0.38	7.27E-05
ko01110	Biosynthesis of secondary metabolites	LOC_Os12g43130	Phytoene synthase 2	Up	2.97	4.38E-05
		LOC_Os11g10520	Alcohol dehydrogenase 2	Up	7.47	4.39E-12
		LOC_Os09g12660	Glucose-1-phosphate adenylyltransferase small subunit 1	Up	5.46	1.58E-03
		LOC_Os09g15820	\	Up	2.06	3.76E-12
		LOC_Os03g63330	Aspartokinase	Up	2.24	9.46E-04
		LOC_Os04g37619	Zeaxanthin epoxidase	Up	5.68	1.97E-40
		LOC_Os08g33720	\	Up	2.60	1.67E-07
		LOC_Os03g60509	Chalcone--flavanone isomerase	Up	2.02	5.04E-09
		LOC_Os08g39840	Lipoxygenase 7	Up	4.22	7.25E-08
		LOC_Os03g20700	Magnesium-chelatase subunit ChlH	Up	2.09	5.08E-05
		LOC_Os11g41710	Cytochrome P450 family protein	Up	5.80	1.54E-05

LOC_Os03g61130	Phosphoesterase family protein	Up	2.24	2.38E-10
LOC_Os04g40950	Glyceraldehyde-3-phosphate dehydrogenase 2	Up	2.34	3.90E-13
LOC_Os11g41680	Cytochrome P450 family protein	Up	8.91	2.17E-09
LOC_Os11g32650	Chalcone synthase 1	Up	17.16	2.89E-10
LOC_Os05g50550	Solanesyl-diphosphate synthase 2	Up	4.11	2.25E-06
LOC_Os09g10340	\	Up	9.24	1.34E-03
LOC_Os12g42884	5-methyltetrahydropteroyltriglutamate--homocysteine methyltransferase 2	Up	2.29	1.92E-07
LOC_Os09g25150	\	Up	2.08	6.50E-05
LOC_Os03g51080	Glutamate decarboxylase	Up	3.00	6.56E-05
LOC_Os03g52170	4-hydroxy-3-methylbut-2-enyl diphosphate reductase	Up	2.64	5.22E-10
LOC_Os07g48050	\	Up	2.88	1.12E-06
LOC_Os12g08810	VTC2	Up	7.90	1.21E-14
LOC_Os11g42510	Tyrosine/nicotianamine aminotransferases family protein	Up	3.45	1.11E-03
LOC_Os10g29470	Probable cinnamyl alcohol dehydrogenase 3	Up	2.25	7.89E-04
LOC_Os04g43390	Beta-glucosidase 16	Up	2.93	1.15E-03
LOC_Os10g17260	Flavonoid 3'-monooxygenase CYP75B3	Up	7.15	1.62E-04
LOC_Os06g10280	\	Up	4.90	3.38E-04
LOC_Os04g52130	Oxygen-dependent coproporphyrinogen-III oxidase	Down	0.46	5.01E-22
LOC_Os12g08260	2-oxoisovalerate dehydrogenase alpha subunit, mitochondrial	Down	0.49	3.31E-04
LOC_Os07g15190	\	Down	0.39	1.99E-03
LOC_Os03g53860	Glycosyl hydrolase family 3 N terminal domain containing protein	Down	0.05	3.42E-14
LOC_Os06g04150	\	Down	0.42	3.35E-30
LOC_Os12g29760	Oxidoreductase, aldo/keto reductase family protein	Down	0.45	6.97E-06

		LOC_Os03g45230	\	Down	0.09	3.59E-06
		LOC_Os07g40690	\	Down	0.18	2.47E-12
		LOC_Os12g35580	Riboflavin synthase	Down	0.43	1.24E-06
		LOC_Os05g48200	Glutamate synthase 2	Down	0.33	2.52E-05
		LOC_Os10g28200	GDP-mannose 3,5-epimerase 1	Down	0.35	3.24E-19
		LOC_Os09g23550	Probable cinnamyl alcohol dehydrogenase 8C	Down	0.43	4.34E-14
		LOC_Os10g02480	NADH-dependent oxidoreductase 2	Down	0.47	5.72E-07
		LOC_Os04g42870	\	Down	0.46	6.98E-04
		LOC_Os12g36194	Nucleoside diphosphate kinase	Down	0.45	8.32E-09
ko00061	Fatty acid biosynthesis	LOC_Os05g04170	\	Up	2.87	9.92E-04
		LOC_Os07g37420	\	Up	2.67	5.29E-09
		LOC_Os04g36800	\	Down	0.41	1.49E-08
ko04530	Tight junction	LOC_Os05g36290	\	Up	2.67	1.87E-03
		LOC_Os04g43710	\	Down	0.28	1.76E-09
ko00906	Carotenoid biosynthesis	LOC_Os12g43130	Phytoene synthase 2	Up	2.97	4.38E-05
		LOC_Os04g37619	Zeaxanthin epoxidase	Up	5.68	1.97E-40

KEGG, Kyoto encyclopedia of genes and genomes (KEGG); Fold change, ratio of fragments per kilobase per million calculated based on transcriptome data between zebra-green striped in leaves and zebra-yellow striped in leaves.
